# Cytotoxic and Antimetastatic Activity of Hesperetin and Doxorubicin Combination Toward Her2 Expressing Breast Cancer Cells

**DOI:** 10.31557/APJCP.2020.21.5.1259

**Published:** 2020-05

**Authors:** Ika Putri Nurhayati, Annisa Khumaira, Gagas Pradani Nur Ilmawati, Edy Meiyanto, Adam Hermawan

**Affiliations:** 1 *Cancer Chemoprevention Research Center, Faculty of Pharmacy, Universitas Gadjah Mada, Indonesia. *; 2 *Department of Pharmacy, Faculty of Medicine, Universitas Brawijaya, Indonesia. *; 3 *Department of Pharmaceutical Chemistry, Faculty of Pharmacy. Universitas Gadjah Mada, Indonesia. *

**Keywords:** Hesperetin, doxorubicin, MCF-7/HER2, cell cycle, metastasis

## Abstract

**Objective:**

This study aimed to explore Hesperetin (Hst) potency as a co-chemotherapeutics agent combined with Doxorubicin (Dox), particularly cytotoxic and antimetastasis effects toward MCF-7/HER2 cells.

**Methods:**

The cytotoxic effects were measured under MTT assay. The flowcytometry analysis was used to examine the cell cycle modulation and apoptosis evidence, while the effect of migration was assayed by scratch wound healing assay. Western blotting and gelatin zymography were carried out to examine the expression level of proteins, HER2, and Rac1.

**Results:**

Under MTT assay, Hst and Dox exhibited to decrease cell viability in a dose-dependent manner with the IC_50_ value of 377 and 0,8 µM, respectively. The combination of Hst and Dox at the respective doses of 95 and 0,2 µM showed a synergistic effect with the combination index of 0,63. Flow cytometry analysis of Hst-Dox revealed that those compounds caused cell cycle arrest at the G2/M phase and induced apoptosis. Hst also decreased HER2 and Rac1 expression, as shown by western blot. Hst inhibited lamellipodia formation and cell migration, as indicated by microscopic observation and wound healing scratch assay. The antimetastatic activity of Hst was associated with the reduction of Rac1 and MMP9 expression as measured by gelatine zymography assay.

**Conclusion:**

These results indicated that the combination of Hst and Dox-induced cell cycle arrest, apoptosis, decreased HER2, Rac1, MMP9 expression, and cell migration. Thus, Hst may have the potential to be developed as a co-chemotherapeutic agent combined with doxorubicin toward HER2 overexpressing breast cancer cells.

## Introduction

HER2 is one of epidermal growth factor receptor family that initiates various signaling pathways, including cell proliferation, survival, differentiation, angiogenesis, and invasion. *HER2* amplification leads to *HER2* overexpression, which found in various types of cancers, such as breast, gastric, esophageal, ovarian, and endometrial cancers (Iqbal and Iqbal, 2014). Furthermore, *HER2* overexpression promotes chemoresistance and increases metastasis potency (De Potter, 1994; Knuefermann et al., 2003). Therefore, HER2 becomes one of the targeted therapy in cancer. Even more, some efforts in this issue have been attempted and resulted in some new findings, such as antibody and small molecules targeted-HER2, but it remains some disadvantages, such as developing resistance of cancer and relatively expensive. Thus the new chemotherapeutic agents targeted to HER2 still need to be developed.

On the other hand, doxorubicin, which is the drug of choice for breast cancer treatment, remains some problems that have to be paid serious attention. Prolonged use of doxorubicin causes several side effects, including toxicity and drug resistance (Tacar et al., 2013). Doxorubicin, the drug of choice in breast cancer, induces metastasis through activation of the TGFβ signaling pathway to trigger Epithelial-mesenchymal transition (EMT) (Bandyopadhyay et al., 2010). This phenomenon leads to a decrease in the efficacy of chemotherapy. Thus, further study needs to be conducted to explore the co-chemotherapeutic agent that combined with doxorubicin in order to increase efficacy and decrease toxicity. In this study, hesperetin, a citrus flavanone mostly found in oranges and lemons, will be explored the anticancer potency, moreover combined with doxorubicin.

Hesperetin has various pharmacological activities, such as antioxidant (Cho, 2006), inhibitor aromatase (Ye et al., 2012), and cytotoxic toward MCF-7 cell (Choi, 2007), MDA-MB 231 (Yang et al., 2012), and HeLa Cells (Alshatwi et al., 2013). Based on these reports, hesperetin tended to increase the sensitivity of MCF-7/Dox cells toward doxorubicin. Hesperetin inhibits Pgp overexpression and decreases resistance to doxorubicin (Meiyanto et al., 2012; Sarmoko et al., 2014). On the other study, hesperetin also showed potential as an HER-tyrosine kinase inhibitor on SKBR3 breast cancer cells (Chandrika et al., 2016). Furthermore, hesperetin also inhibited the TGF-β signaling pathway and phosphorylation of Smad3, thus decreased metastasis (Yang et al., 2012).

In this study, we found that hesperetin exhibited a synergistic effect with doxorubicin toward HER2 overexpressed breast cancer cell, MCF-7/HER2. The combination of Dox and Hst performed cytotoxic activity through cell cycle modulation and apoptosis induction. Hesperetin also decreased cell metastasis by inhibiting lamellipodia formation and cell migration. These effects were related to the combined activity on decreasing expression of HER2, Rac1, and MMP9. Based on those results, the combination of hesperetin and doxorubicin exhibited potency to kill and inhibited metastasis of HER2 expressing breast cancer cells. 

## Materials and Methods

*Cell culture*

MCF-7/HER2 and MCF-7/EV cell line were kindly given from Prof. Dr. Yoshio Inouye through Prof. Dr. Mashashi Kawaichi (NAIST, Japan) and were cultured in a CO2 incubator (37C) with DMEM (Gibco) supplemented with 10% FBS (Sigma), 1.5% Penicillin-Streptomycin (Sigma), and 0.5% Fungizone (Sigma). Subculture was performed using trypsin-EDTA (Gibco) for cell detachment. Samples used were Hesperetin (Sigma) and Doxorubicin (Sigma).

*MTT Cytotoxicity Assay*

The cytotoxicity assay was performed using the MTT assay. The 2x10^3^ MCF-7/HER2 cells were seeded into each well on 96 well-plate. On the next day, cells were treated with Hst and Dox at various concentrations for 24 h. Cells were added with 100 uL of 0.5 mg/mL 3-(4,5-dimethylthiazol-2-yl)-2,5-diphenyltetrazolium (MTT) reagent (Biovision) and incubated for 2-4 h. At the end of the incubation period, an SDS stopper solution containing 0,01N HCl was added to each well. The absorbance was measured by ELISA reader at 595 nm. The percentage of cell viability was calculated from absorbance data. The IC_50_ value was obtained from linear regression between the concentration of sample and cell viability percentage. For cytotoxic combination assay, combination index and isobologram were analyzed with CompuSyn^®^ software based on the Chou-Talalay method (Blumenthal et al., 2005). 

*Cell Cycle Assay*

The 8x10^4^ MCF-7/HER2 cells each well were seeded in a 6-well plate. Each well was treated with Hst, Dox, and the combination at a certain concentration for 24 h. Cells were harvested using trypsin-EDTA, collected, and then stained with the BD Cycletest Plus DNA kit (BD) according to manufacturer instruction. Cells suspension was transferred to a cytometric tube and analyzed further using flow cytometry (BD FACS Calibur, BD Bioscience) and Cell Quest software. 

*Apoptosis Assay*

The 8x10^4^ MCF-7/HER2 cells were seeded in 6 well-plate and treated with Hst, Dox, and its combination for 24 h. Cells were harvested using trypsin-EDTA, collected, and stained using FTIC Annexin V (BD) staining kit according to manufacturer instructions. The cell suspension was analyzed using flow cytometry (BD FACS Calibur, BD Bioscience). 

*Lamellipodia formation and Scratch Wound Healing Assay*

The 7x10^4^ MCF-7/HER2 cells were seeded into a 6-well plate and treated with 95 µM Hst, 10 nM Dox, and the combination on a subsequent day. Lamellipodia formation was observed at 0, 24, and 48 h after treatment using the inverted microscope (100x magnification). Cell migration inhibition was tested using a scratch wound healing method. The 8.5x10^4^ MCF-7/HER2 cells were seeded into 24-well plate and incubated for 24 h. Cell starvation was performed by incubating cells on medium containing 0.5% FBS. Cells were scratched using a sterile yellow tip to and treated with Hst, Dox, and its combination. Observation and documentation to each scratched area were done at 0, 16, 24, and 42 h after treatment and then further analyzed using ImageJ software. 

*Gelatin Zymography*

The expression of MMP9 was performed using gelatin zymography. SDS-PAGE supplemented with 0.1% gelatin was used in this method. Following electrophoresis, a gel was incubated with 2% of Triton-X 100 (Merck) in water for 30 min at room temperature, and the solution was removed from gels. Furthermore, incubating buffer (consist of 40 mM Tris-HCl pH 8, 10 mM CaCl_2_, 0.02% NaN_3_) was added to the gel and incubated again at 37°C for 18-20 h. Then, the gel was stained using Coomassie Brilliant Blue R-250 solution and destained until a clear band with the blue background was observed. Those bands were documented and analyzed using ImageJ software.

*Western Blotting*

Expression of HER2 and Rac1 were observed by western blot. Cells were treated with Hst, Dox, and combination at a certain concentration for 24 h. Cells were lysed in RIPA buffer (Tris HCl pH 7.6 25 mM, NP40 1%, Na deoxycholate 1%, NaCl 150 mM, SDS 0,1%), PMSF 100 mM, NaF 200 mM, and proteinase inhibitor. The lysate was centrifuged to separate the cell pellet and supernatant. Protein concentration was determined using the Bradford method. The sample was loaded into SDS-PAGE gel and run for electrophoresis. After electrophoresis, the protein was transferred onto PVDF membranes (Merck). Blocking was done by incubated PVDF membrane in net gelatin for 1 h followed by incubation using a primary antibody (HER2 (Santa Cruz^®^ cat.52349) and Rac1 (Cell Signalling^®^ cat. 22475)) overnight at 4°C. The step was continued with secondary anti-mouse HRP conjugated antibody (anti-mouse IgG-HRP Santa Cruz^®^) incubation for 1 h after washed with NET-gelatin. A specific band from each protein was examined by adding ECL reagent (GE Life Science) to the membrane and observed using Luminograph (Atto).

## Results

*Effect of Hesperetin and Doxorubicin on Cell Viability of MCF-7/HER2*

**Figure 1:**
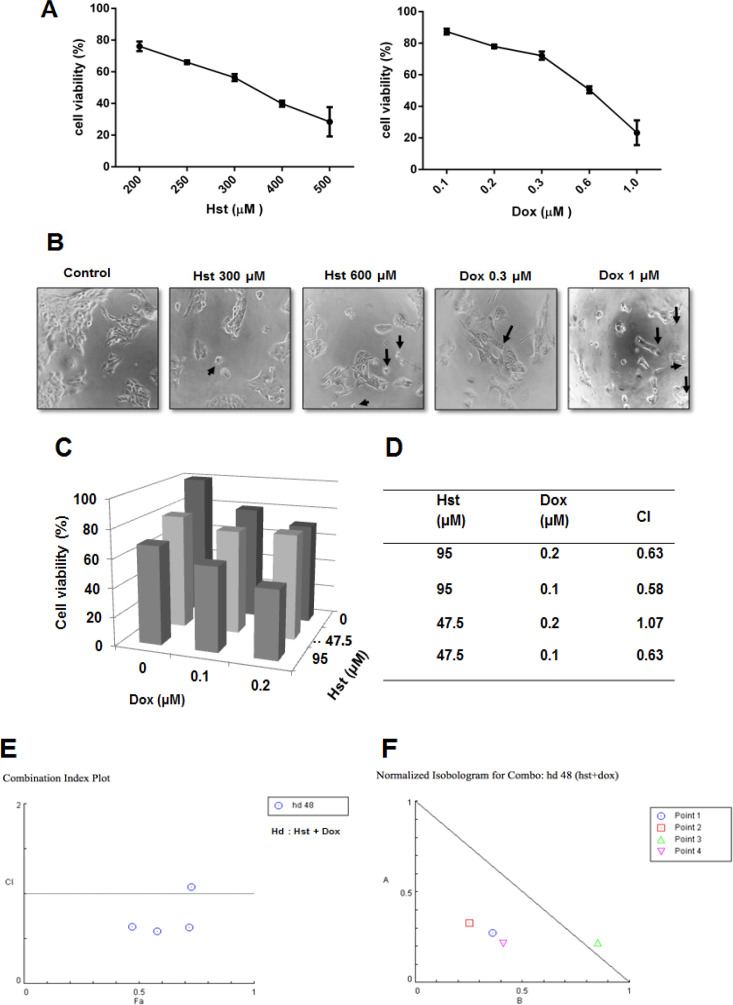
Cytotoxicity of Hst, Dox, and Their Combination on MCF-7/HER2 Cells. The 1.5x10^3^ MCF-7/HER2 cells were seeded into 96 well plates and treated with Hst, Dox, and the combination on the following days. The cytotoxic assay was performed using the MTT method, as described in methods. (A), Percentage of cell viability after a single treatment of Hst and Dox at various concentrations for 24 h. Profiles of cell viability were expressed as mean ± SD from 3 independent experiments; (B), Change of cells morphology after Hst and Dox treatment for 24 h. Arrows indicate changes in each cell morphology; (C), Effect of the combination of Hst and Dox treatment on cell viability; (D), Index combination value; (E), Combination index plot, and (F), Isobologram for a combination of Hst and Dox measured by CompuSyn® software based on Chou-Talalay method

The effect of hesperetin and doxorubicin on the viability of MCF-7/HER2 cells was measured by MTT assay. A single treatment of hesperetin and doxorubicin at various concentrations decreased cell viability in a dose-dependent manner. IC_50_ of hesperetin and doxorubicin were 377 and 0.8 µM, respectively (Figure 1A). Both compounds also changed cell morphology, such as cell shrinkage (Figure 1B). Hesperetin, in combination with doxorubicin, increased cytotoxic activity compared to single treatment of each compound. The CI value of those combinations was measured using CompuSyn^®^ software. An optimum concentration of Hst 95 μM and Dox 0.2 μM combination for 24 h decreased cell viability up to 42.54% (Figure 1C). CI value obtained at those concentrations is 0.63 and categorized as a combination with synergistic effect (Figure 1D). A combination index plot showed that one combination of treatment exhibited an additive effect with CI value nearly 1 (Figure 1E). The isobologram, (at fa=0.5, 0.75 and 0.9) showed that three isoboles of combined Hst and Dox were located to the left of the curve, which indicates that Hst had a synergistic effect with Dox (Figure 1F). Moreover, one isobole of the combinations that fall slightly above the line of additivity represented additive effects. A further assay was needed to examine whether the reduction of cell viability caused by apoptosis or cell cycle modulation.

*Effect of Hesperetin and Doxorubicin on Cell Cycle and HER2 Expression*

**Figure 2:**
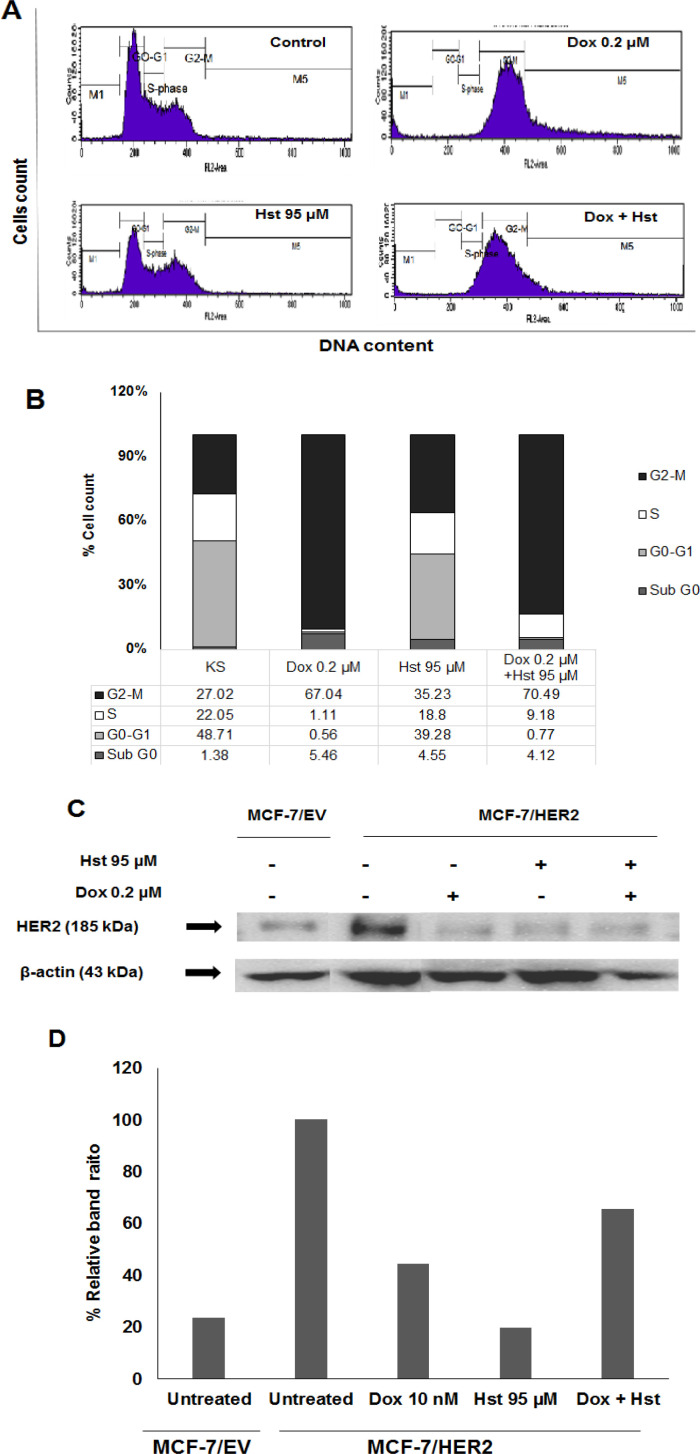
Effect of Hst and Dox on Cell Cycle and HER2 Expression of MCF-7/HER2. MCF-7/HER2 was seeded into a 6-well plate, added with Hst Dox, and the Combination and Incubated for 24 h. Cells were harvested and added with PI reagent and analyzed using a flow cytometer. (A), Cell distribution profile of (i) cell control (ii) Dox 0.2 μM (iii) Hst 95 μM dan (iv) combination Hst and Dox; (B), Percentage of the cell population in each phase after various treatments; (C), HER2 expression was examined using western blot. Band visualization was obtained using Luminograph; (D), Band obtained were quantified using ImageJ software showed an intensity ratio of HER2 and ß-actin after treatment with Hst, Dox, and the combination showed that the treatment decreased HER2 expression

The effect of hesperetin and doxorubicin combination on cell cycle modulation was performed by PI staining and analyzed using a flow cytometer. A single treatment of 95 μM Hst increased cell accumulation at G2/M phase 35.23%; meanwhile, doxorubicin also increases cell accumulation 67.04%. A combination of 95 Hst and 0.2 μM Dox also induced cell cycle arrest at the G2/M phase 70.49%. Cell accumulation at S phase also elevated during combination treatment (Figure 2B). Accumulation on the G2/M phase was associated with HER-2 role in regulating the cell cycle. HER2 expression was examined using western blot to comprehend whether single or combination treatment altered HER2 expression. HER2 expression in MCF-7/HER2 cells was higher than MCF-7/EV indicated that MCF-7/HER2 cells undergo HER2 overexpression. A single treatment of Dox 0,2 µM and Hst 95 µM decreased HER2 expression, but the Hst effect was stronger than Dox (Figure 2D). Combination treatment also decreases HER2 expression, but this effect was lower than a single treatment. Furthermore, the Hst effect on cell apoptosis was observed using flow cytometry.

*Effect of Hesperetin and Doxorubicin on Apoptosis*

**Figure 3:**
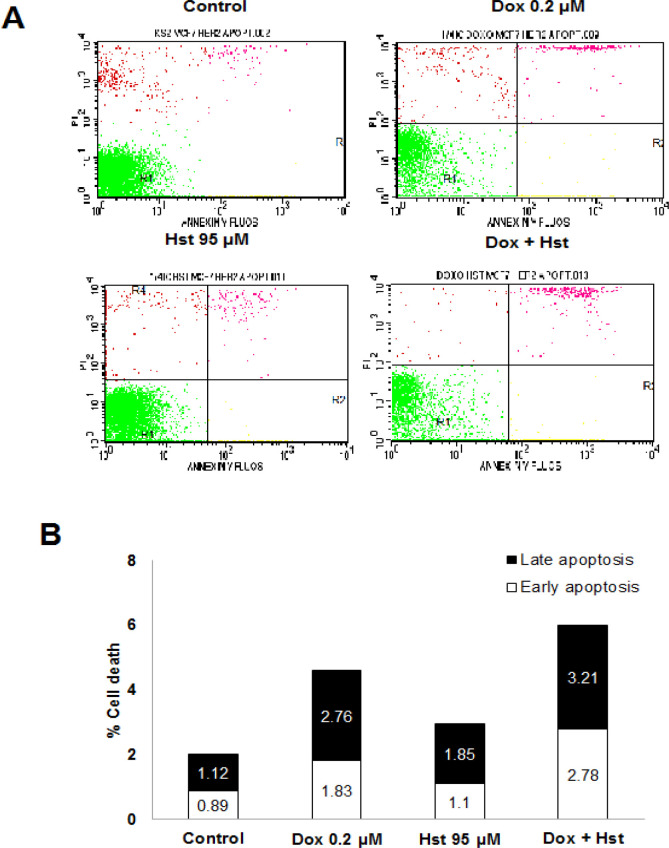
Effect of Hst, Dox, and Combination on Cell Apoptosis. The 1.5x10^5^ cells were seeded into a 6-well plate and incubated for 24 hours. The next day, each well was treated with Hst, Dox, and the combination for 24 hours. Furthermore, samples were stained with Annexin V and propidium iodide (PI) and analyzed using flow cytometry. (A), Profile of cell distribution after treatment consists of living cells (bottom-left), cells undergo early apoptosis (bottom-right), late apoptosis (upper-right), and necrosis (upper-left); (B), Percentage of cells undergo early and late apoptosis

Effect of Hst and Dox on cell apoptosis was observed using flow cytometry after sample preparation and staining using annexin V and PI reagent. Based on this method, cells undergo early apoptosis, late apoptosis, and necrosis could be distinguished. Dox 0.2 μM raised cell apoptosis 4.95% compared to normal cells. A single treatment of Hst 95 μM increased cell apoptosis 2.95%; meanwhile, the combination increased by 5.99% (Figure 3B). Combination treatment elevated cell apoptosis compared to single treatment of Dox and also untreated cell. Despite that, the number of living cells was high, which higher than 80%. Based on the cytotoxic assay, the combination of Hst and Dox-induced cell cycle arrest and apoptosis. Further study needed to be conducted to explore Hst activity as antimetastasis toward HER2, overexpressed breast cancer cells.

*Effect of Hesperetin on Lamellipodia Formation and Cells Migration*

Dox, one of the chosen drugs in breast cancer treatment, induce EMT formation leading to innovation. Furthermore, Dox-induced cell migration to other tissues. The antimetastatic effect of Hst was observed to examine its ability to inhibit cell invasion and migration toward MCF-7/HER2 using an inverted microscope with 100x magnification at a particular interval of time. We discovered that Dox 10 nM led to change the morphology of the cells compared to untreated cells. Lamellipodia elongation also observed after Dox treatment. Even though 95 µM Hst treatment did not alter cell morphology, the combination with Dox reduced lamellipodia elongation, which also observed in single Dox treatment. Collectively, Dox and Hst combination showed potent inhibition of lamellipodia formation induced by Dox.

**Figure 4:**
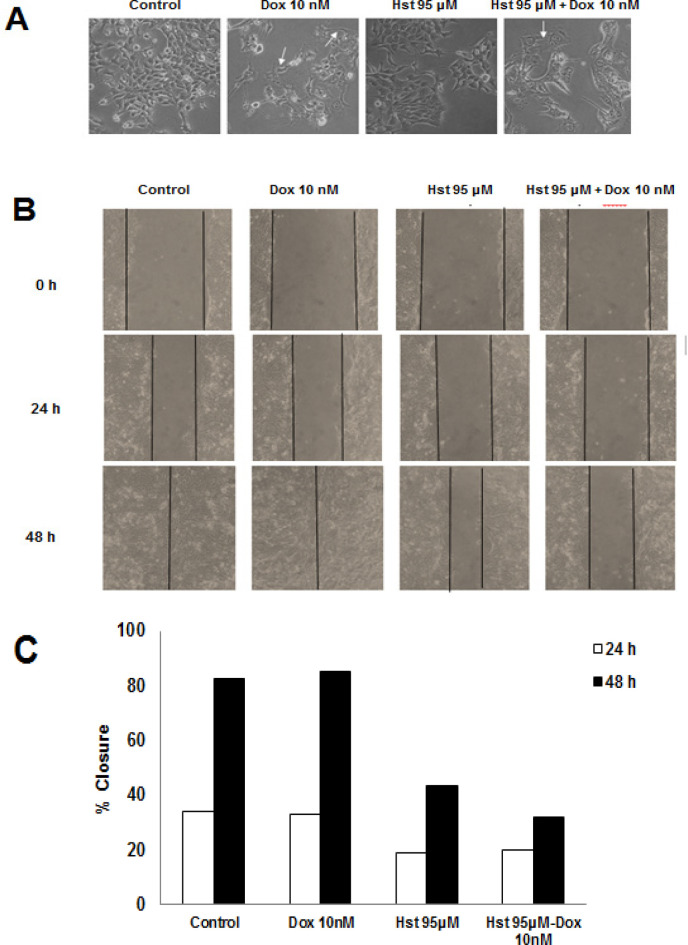
Effect of Hst, Dox, and Their Combination on Lamellipodia Formation and Cells Migration. An inverted microscope was used to observe lamellipodia formation after treatment with 100x magnification. (A), Morphological change after Hst, Dox, and the combination treatment. White arrow showed lamellipodia of a cell, and observation was done at a certain interval of time; (B), Inhibition of cell migration induced by Dox 10 nM was examined using scratch wound healing assay. Scratch area was observed at a certain time using an inverted microscope at 100x magnification; (C), Percent closure after Hst, Dox, and the combination treatment at 24 and 48 h measured using ImageJ software

Inhibition of cell migration was performed by scratch wound healing assay. Control group and Dox 10 nM showed a high percent closure. A single treatment of 95 µM Hst for 24 h exhibited 18% closure meanwhile 48 h treatment was 43%. The combination showed 19% closure on 24 h treatment and 32% on 48 h (Figure 4). Those results indicate that Hst either on single or combination treatment inhibited cell migration. Further research to explore the effect of Hst on the protein involved in metastasis needs to be conducted. In this study, MMP9 and Rac1 expression will be observed using gelatin zymography and western blot method, respectively.

*Hesperetin and Doxorubicin Effect on Rac1 and MMP9 Expression*

**Figure 5:**
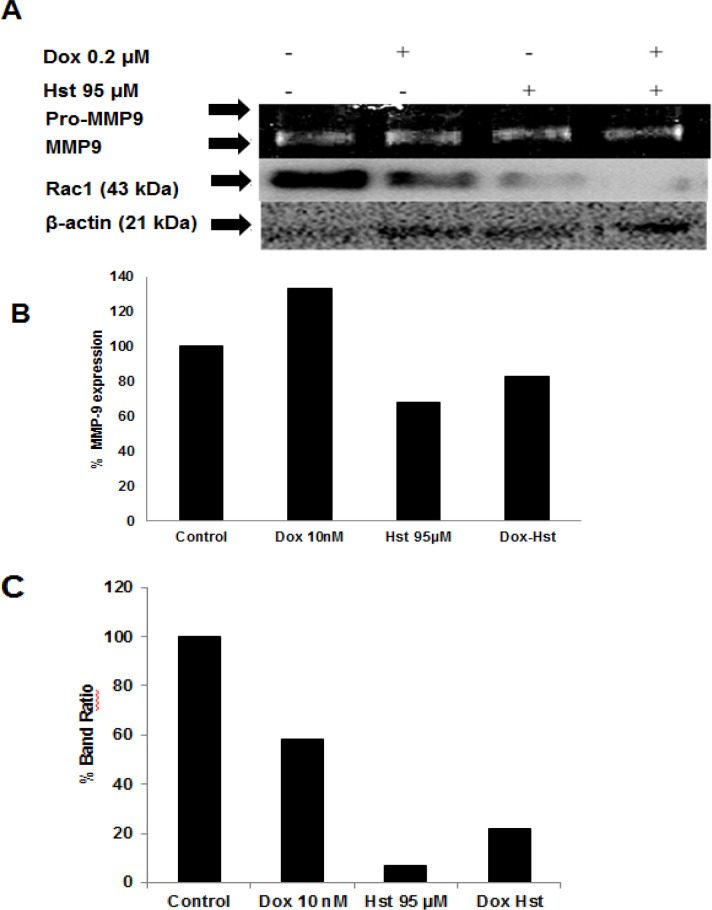
Effect of Hst and Dox on Rac1 and MMP9 Expression. MMP9 expression was performed using gelatin zymography, meanwhile, Rac1 using western blot. (A), Band of Rac1 and MMP-9 after Hst, Dox, and combination treatment. The band was quantified using ImageJ software; (B), A percentage of MMP9 expression relative to control cells; (C), Percentage of Band Ratio of Rac1 between treatment and control cells

The metastasis process involved the Rac1 protein that plays a role in lamellipodia formation and MMP9 enzyme that degrade the extracellular matrix. Expression of Rac1 was performed by western blot meanwhile MMP9 by gelatin zymography. MMP enzyme degraded gelatin embedded in SDS-PAGE gell led to a clear band with a dark blue background. The area of each band then quantified using ImageJ software. A single treatment of Dox 10 nM enhanced MMP9 expression compared to control. When Hst, combined with Dox, MMP9 expression decreased by 83% (Figure 5). These results show that Dox increased MMP9 expression; meanwhile, the combination of Dox and Hst decreased MMP9 expression. Rac1 is a protein involved in lamellipodia formation leading to cell migration. Untreated cells express high Rac1; meanwhile, Dox and Hst treatment decreased Rac1 expression. The combination of Dox and Hst also decreased Rac1 expression, but it was lower than single treatment (Figure 5). Thus, the combination of Hst and Dox decreased expression of a protein that plays a role in cancer metastasis, which is MMP9 and Rac1.

## Discussion

Resistance phenomenon after chemotherapy treatment triggered the development of novel compounds that combined with chemotherapeutic agents, called co-chemotherapy. An active compound from the medicinal plant could be developed as a co-chemotherapeutic agent, such as hesperetin. Hesperetin exhibit various pharmacological activities, for example, antioxidant, cytotoxicity toward several cell cancer line, induce apoptosis, and inhibit angiogenesis (Cho, 2006; Choi, 2007; Kim, 2014). In this study, hesperetin has been explored as an anticancer agent toward HER2 overexpressed cancer cells to explore its cytotoxicity and antimetastasis potency combined with doxorubicin.

Hesperetin exhibits cytotoxicity in a dose-dependent manner on MCF-7/HER2 cells. Cell viability decreased with the higher dose of hesperetin. Hesperetin also showed time-dependent cytotoxic activity toward MCF-7 cells (Choi, 2007). A combination of hesperetin and doxorubicin reached optimum effect at the dose 95 µM and 0.2 µM, respectively. That combination exhibited a synergistic effect based on the combination index value and isobologram analysis. The previous study confirmed that hesperetin enhanced cell sensitivity to doxorubicin with optimal doses of 95 and 0.23 µM, respectively (Sarmoko et al., 2014).

Furthermore, to explore the mechanism of action of hesperetin to decrease cell viability, cell cycle assay and apoptosis was performed using flow cytometry. Dox at 0.2 µM increased cell accumulation at G2/M and S phase. It was already understood that doxorubicin inhibits topoisomerase II enzyme, which represses gene transcription and leads to DNA break with p53 protein stabilization, thus inhibit cells from entering mitosis, however leading to arrest at G2/M phase (Taylor and Stark, 2001; Yang et al., 2012). Hesperetin with a concentration of 95 µM and the combination with 0.2 µM doxorubicin-induced cell cycle arrest at the G2/M phase. HER2 signaling activates PI3K that needed in cell cycle progression, especially G2/M (Liang and Slingerland, 2003). To confirm whether that activity was related to HER2 protein on cell cycle regulation, the expression of HER2 was observed using western blot. Hesperetin and the combination with doxorubicin decreased HER2 expression. In silico and in vitro studies toward HER2 kinase activity showed that hesperetin interacts with ATP-binding site of HER2 tyrosine kinase. The result indicated hesperetin has the potential to be further explored as an inhibitor of HER2-tyrosine kinase (Chandrika et al., 2016).

In this study, the combination of hesperetin and doxorubicin-induced MCF-7/HER2 cell apoptosis despite this enhancement was not significant. Hesperetin induced apoptosis on MCF7 cells by decrease Bcl2 and increase Bax after 72 h treatment (Choi, 2007). Hesperetin also induces apoptosis on SKBR3 cells by the time-dependent manner, in which the apoptosis effect was observed after 36 h of treatment (Chandrika et al., 2016). Thus, further study needs to be conducted with a prolonged time of treatment.

Doxorubicin, one of the drugs of choice in breast cancer, can activate TGFβ signaling on a cancer cell, leading to increased cancer malignancy. Activation of TGFβ induces EMT caused increasing cell motility and invasiveness (Bandyopadhyay et al., 2010). In this study, hesperetin activity as an anticancer was examined by test its ability to inhibit cell migration and invasion. A combination of hesperetin and doxorubicin reduced lamellipodia formation induced by 10 nM doxorubicin without affecting cell viability. Lamellipodia formation involved Rac1 protein that plays a role in the reassembly of actin needed in lamellipodia formation (Pradip et al., 2017). Rac1 protein expression was observed using the western blot method. Both single and combination treatment of hesperetin and doxorubicin decreased Rac1 expression. Cell motility mediated by Rac-1 via TGF-β induction needs HER2 activation at a certain level (Wang et al., 2006). Thus, these results indicated that the reduction of Rac1 expression related to the reduction of HER2 expression. Inhibition of cell migration by hesperetin also examined by scratch wound healing assay. Hesperetin inhibited cell migration both in single treatment and combination with doxorubicin. These results affected by hesperetin activity as an inhibitor of the TGF-β signaling pathway. Hesperetin reduced the binding of TGF-β and the receptor and phosphorylation of Smad that involved in the regulation of transcription of the gene that plays a role in the EMT process. TGF-β also activates Rhoa and Rac1 that contribute to EMT and cell motility (Wang et al., 2006). Cancer cell invasion involved the MMP enzyme that degrades the extracellular protein matrix by breaking protein bonding leading to cell migration. In this study, doxorubicin increased MMP9 expression, whereas hesperetin both as single meanwhile, the combination treatment decreased MMP9 enzyme expression. HER2 protein also plays a role in regulating MMP9 expression on the transcriptional level (Shan et al., 2015). Hesperetin activity in decreasing MMP9 activity seemed to related to a decrease of HER2 expression.

In conclusion, taken together, the combination of Hst and Dox inhibited cancer cell’s growth and metastasis through cell cycle arrest, apoptosis, reduce cell migration, decrease HER2, Rac1, and MMP9 expression. Therefore, Hst may have the potential to be developed as a co-chemotherapeutic agent.
